# Transcriptome Analysis Reveals Candidate Genes Associated with Leaf Etiolation of a Cytoplasmic Male Sterility Line in Chinese Cabbage (*Brassica Rapa* L. ssp. *Pekinensis*)

**DOI:** 10.3390/ijms19040922

**Published:** 2018-03-21

**Authors:** Fei Xie, Jia-Lan Yuan, Yi-Xiao Li, Can-Jie Wang, Hong-Yu Tang, Jun-Hui Xia, Qing-Yong Yang, Zheng-Jie Wan

**Affiliations:** 1 Key Laboratory of Horticultural Plant Biology, Ministry of Education, College of Horticulture and Forestry Sciences, Huazhong Agricultural University, Wuhan 430070, China; xf507@webmail.hzau.edu.cn (F.X.); lyxzzl@webmail.hzau.edu.cn (Y.-X.L.); wangcanjie@webmail.hzau.edu.cn (C.-J.W.); tanghongyu@webmail.hzau.edu.cn (H.-Y.T.); xiajh@mail.hzau.edu.cn (J.-H.X.); 2 Hubei Key Laboratory of Agricultural Bioinformatics, College of Informatics, Huazhong Agricultural University, Wuhan 430070, China; yuanjl@webmail.hzau.edu.cn (J.-L.Y.); yqy@mail.hzau.edu.cn (Q.-Y.Y.)

**Keywords:** Chinese cabbage, cytoplasmic male sterility, leaf etiolation, chlorophyll synthesis, chloroplast development

## Abstract

Cytoplasmic male sterility (CMS) is universally utilized in cruciferous vegetables. However, the Chinese cabbage *hau* CMS lines, obtained by interspecific hybridization and multiple backcrosses of the *Brassica juncea* (*B. juncea*) CMS line and Chinese cabbage, show obvious leaf etiolation, and the molecular mechanism of etiolation remains elusive. Here, the ultrastructural and phenotypic features of leaves from the Chinese cabbage CMS line 1409A and maintainer line 1409B are analyzed. The results show that chloroplasts of 1409A exhibit abnormal morphology and distribution. Next, RNA-sequencing (RNA-Seq) is used to identify 485 differentially expressed genes (DEGs) between 1409A and 1409B, and 189 up-regulated genes and 296 down-regulated genes are found. Genes that affect chloroplasts development, such as *GLK1* and *GLK2*, and chlorophyll biosynthesis, such as *PORB*, are included in the down-regulated DEGs. Quantitative real-time PCR (qRT-PCR) analysis validate that the expression levels of these genes are significantly lower in 1409A than in 1409B. Taken together, these results demonstrate that leaf etiolation is markedly affected by chloroplast development and pigment biosynthesis. This study provides an effective foundation for research on the molecular mechanisms of leaf etiolation of the *hau* CMS line in Chinese cabbage (*Brassica rapa* L. ssp. *pekinensis*).

## 1. Introduction

Chinese cabbage (*Brassica rapa* L. ssp. *pekinensis*; 2*n* = 20) belongs to the large *Brassicaceae* family and is one of the most widely planted leaf vegetables in China, where it originated. This crop is very rich in germplasm resources, and commercial producers currently have year-round production. Because Chinese cabbage is a cross-pollination crop with strong heterosis, the current cultivars are almost all F_1_ hybrids [1]. Self-incompatibility and male sterility are effective means to exploit heterosis in cruciferous vegetables; cytoplasmic male sterility (CMS) is one of the most widely used systems [2]. Many CMS types have been selected in cruciferous plants, including *pol* [3], *ogu* [4], *tour* [5], *nap* [6], and *hau* [7]. Among them, *hau* CMS, originally found in *B. juncea*, is very suitable for cruciferous plant breeding because of its stability and complete sterility [8]. Subsequently, male sterility was successfully transferred to Chinese cabbage by interspecific hybridization and multiple-generation backcross. However, the leaves of the CMS plants of Chinese cabbage exhibit a high degree of etiolation, turning almost yellow due to incompatibilities between the *B. juncea* sterile cytoplasm and the *Brassica rapa* (*B. rapa*) nucleus. Therefore, revealing the mechanism of leaf etiolation caused by the interspecific hybridization of *B. juncea* cytoplasmic sterile lines with Chinese cabbage and obtaining *B. rapa* plants with green sterile leaves are breeding goals.

Among several pigments produced in the chloroplast, chlorophyll (Chl) is a dominant component in normal green leaves. In plants, Chl comes from the tetrapyrrole biosynthesis pathway, which occurs primarily in plastids [9]. Chl plays a key role in harvesting and transferring light energy as well as in driving electron transfer into the photochemical reaction centers [10]. Protochlorophyllide oxidoreductase (POR) is a key enzyme for the light-induced greening of etiolated angiosperm plants [11]. This enzyme catalyzes protochlorophyllide (Pchlide) to chlorophyllide (Chlide) conversion [12]. The *Arabidopsis* genome encodes three POR homologs, designated as *PORA, PORB* and *PORC*, which are differentially expressed during plant development [13]. *PORA* and *PORB* genes are strongly expressed early in seedling development. Unlike PORA, the import of PORB into plastids is not affected by the Pchlide content of the plastid, neither in true leaves nor in cotyledons [14]. *PORB* plays an important role in Pchlide homeostasis and greening of etiolated plants. Failure to express functional *PORB* leads to photodynamic damage due to the excess accumulation of free Pchlide molecules, acting as photosensitizers and provoking the generation of singlet oxygen [15].

The quantity, shape and distribution of chloroplasts in the leaf directly affect the leaf color. Thus, the dysfunction of chloroplasts usually results in the loss of leaf green color [16]. Leaf etiolation of mutants is determined by the expression level of the key genes involved in chloroplast development [17]. Several genes affecting chloroplast development have been reported, and their role in leaf etiolation has been identified through studies of leaf mutants in *Arabidopsis thaliana* [18], rice [19], and tomato [20]. Golden 2-like (*GLK*) gene family includes *GLK1* and *GLK2*, which serve as regulation factors adjusting chloroplast development in various plant species, such as *Brassica napus* [21], tomato [22,23], wheat [24] and tobacco [25]. Recently, research on *GLKs* has rapidly increased and shown that *GLKs* control chloroplast development in green and non-green tissues [26]. 

Anthocyanin biosynthesis is crucial for leaf and fruit color. The biosynthetic pathways of anthocyanins have been well characterized, and the corresponding genes have been isolated from various plants [27]. Among key enzymes in anthocyanin biosynthesis are chalcone synthase (CHS), flavanone 3-hydroxylase (F3H), dihydroflavonol 4-reductase (DFR), and anthocyanidin synthase (ANS). [16]. In addition to these biosynthetic genes, coordination with R2R3-type MYELOBLASTOSIS (MYB), basic helix-loop-helix (bHLH) transcription factors (TFs) and WD40-repeat-containing (WDR) proteins and their interactions are also involved in the regulation of anthocyanin biosynthesis at the transcriptional level [28,29,30]. MYB and bHLH are the largest families among plant TFs and are found in all eukaryotes [31]. WDR proteins are strongly conserved in eukaryotes and play vital roles in plant-specific processes, with diversity in their functions in upstream signaling pathways and diversity in their downstream regulatory targets [32]. Moreover, anthocyanin transport affects leaf color. The accepted hypothesis includes the transport processes mediated by the glutathione S-transferase (GST) family [33], the ATP-binding cassette (ABC) [34] and multidrug and toxic compound extrusion (MATE) family [35,36].

Most research on leaf etiolation focuses on mutants. However, interspecific cytoplasmic hybridization in cruciferous plants would also cause leaf etiolation [37,38,39]. The yellowing material in our study, namely, the Chinese cabbage sterile line 1409A, was obtained by interspecific crosses between the recently discovered green normal *hau* CMS line of *B. juncea* and green normal maintainer line 1409B of Chinese cabbage. The etiolated 1409A potentially possesses the incompatibility of the *B. juncea* plastids and the *B. rapa* nucleus.

In this study, the micro- and ultrastructure of the chloroplasts and physiological characteristics of the etiolated CMS line 1409A and green normal maintainer line 1409B in Chinese cabbage are further analyzed. Leaf transcriptomes from 1409A to 1409B in the Chinese cabbage are sequenced, and differently expressed genes (DEGs) in the two groups of tissues are identified. The expression of genes involved in leaf etiolation is validated by qRT-PCR. The primary goal of the research is to fully clarify the gene expression differences between 1409A and 1409B and to explore the molecular mechanisms responsible for leaf etiolation. Genetically improving and overcoming the poor quality traits of the sterile lines to obtain leaf color and growth of normal sterile lines would promote the utilization of the *Brassica juncea hau* CMS line in Chinese cabbage and other *Brassica* vegetables.

## 2. Results

### 2.1. Phenotype and Ultrastructure of the Etiolated and Non-Etiolated Leaves

**Figure 1:**
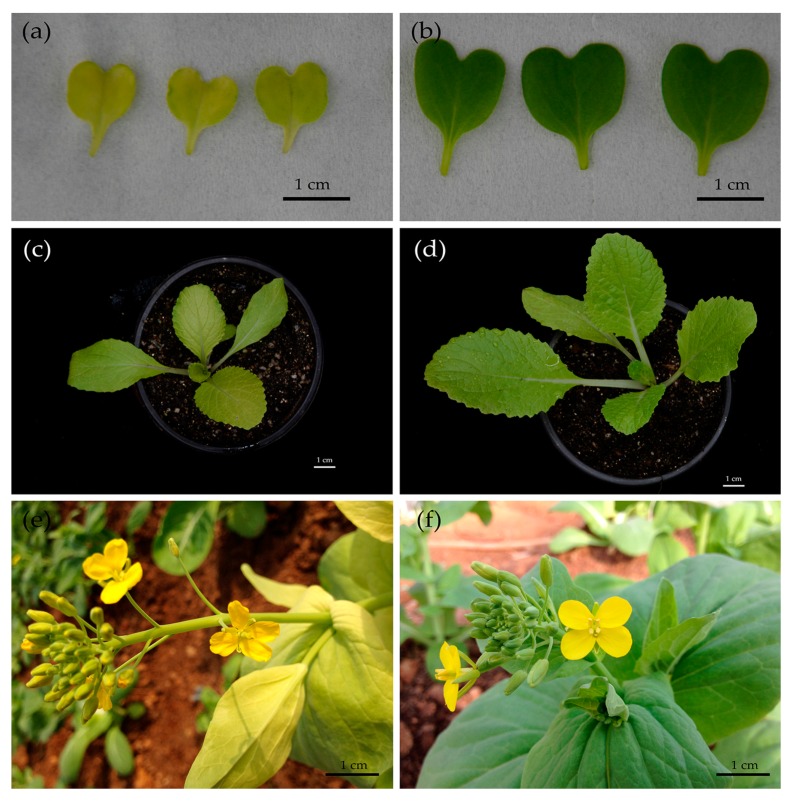
The morphology of 1409A and 1409B in different stages: (**a**,**c**,**e**) 1409A plants in dicotyledonous stage, seedling stage and flowering stage, respectively (Scale bar = 1 cm); (**b**,**d**,**f**) 1409B plants in dicotyledonous stage, seedling stage and flowering stage, respectively (Scale bar = 1 cm).

We obtained the Chinese cabbage CMS line 1409A by interspecific hybridization and backcrosses with the mustard *hau* CMS, which showed an unusual etiolated leaf phenotype compared with its maintainer line 1409B, characterized by typical green leaves (Figure 1). Notably, this leaf color difference between 1409A and 1409B was apparent even in the early stage of the dicotyledonous period (Figure 1a). 

**Figure 2:**
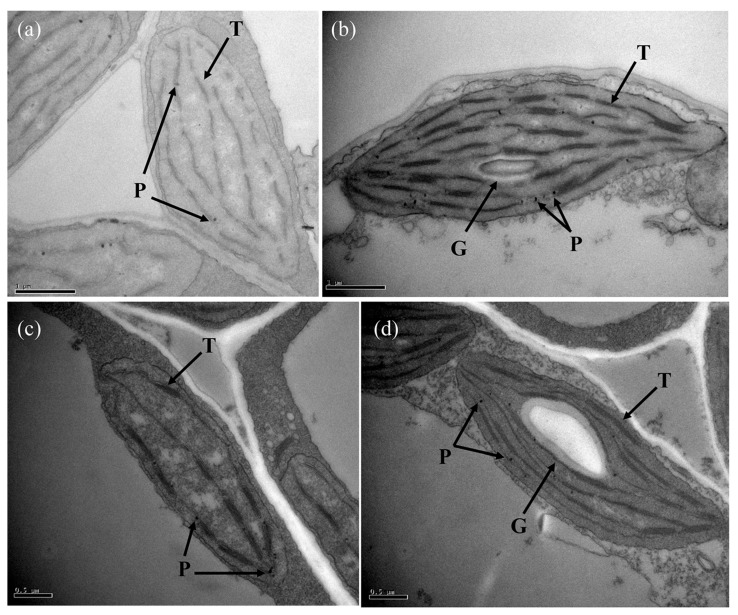
Chloroplast ultrastructure of 1409A and 1409B: (**a**,**c**) chloroplast ultrastructure analysis of 1409A in seedling stage and flowering stage (Scale bar = 1 µm; Scale bar = 0.5 µm); and (**b**,**d**) chloroplast ultrastructure analysis of 1409B in seedling stage and flowering stage (scale bar = 1 µm; scale bar = 0.5 µm). In these pictures, G denotes granulose, P denotes plastoglobuli, and T denotes thylakoid grana. The black arrows were added artificially for visual indication.

Corresponding to the phenotypes, transmission electron microscope (TEM) analysis indicated apparent differences in the ultrastructure of the etiolated and green leaves (Figure 2). The chloroplasts of the etiolated leaves were arrested at the seedling and flowering stages, and they did not develop a mature starch granule, which leads to inanimate plants, and did not have intact grana structures or a clear thylakoid membrane, thus blocking chlorophyll synthesis and photosynthesis. Meanwhile, the membrane system of the etiolated leaves was severely disrupted, and chloroplasts showed signs of cavitation, especially at the flowering stage (Figure 2c).

### 2.2. Content of Main Pigments and Photosynthetic Rate

**Figure 3:**
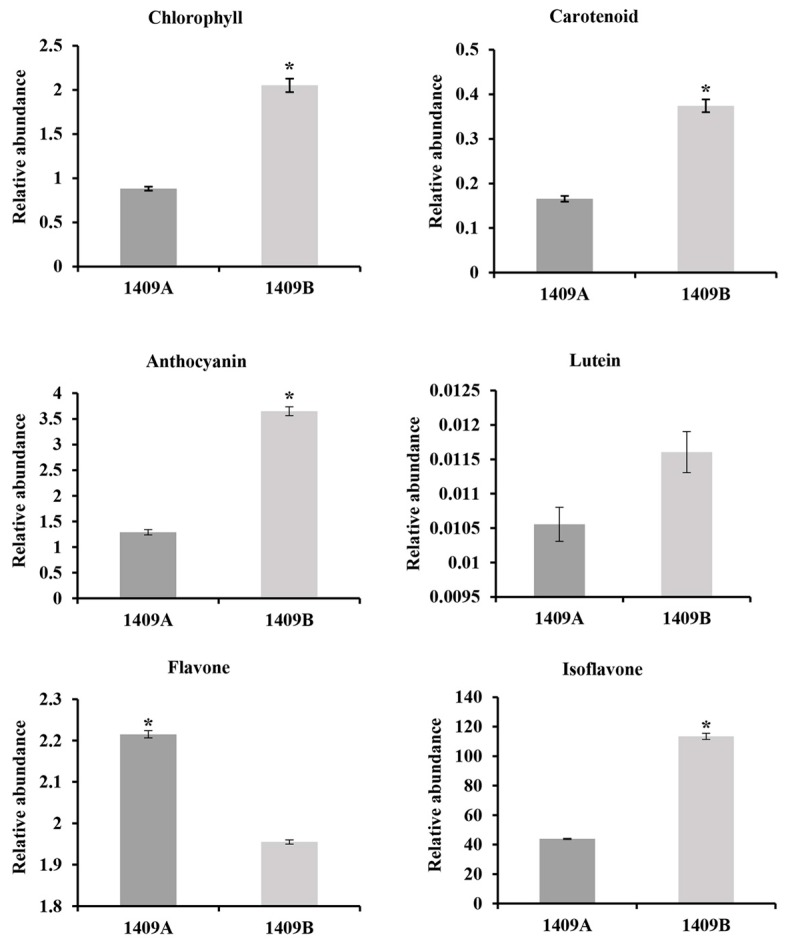
Pigment accumulation in leaves of 1409A and 1409B at the seedling stage. Values are the means ± SE from three biological replicates (*n* = 3). Asterisks indicate the significant differences (* *p* < 0.01) between 1409A and 1409B as determined by Student’s *t* test.

Leaf color is mainly controlled by the content of pigments. To determine which pigments are affected in etiolated leaves, we measured the content of chlorophyll, carotenoid, anthocyanin, lutein, flavone and isoflavone in the leaf of 1409A and 1409B during the seedling stage. As predicted, significant changes were noticed in all the above pigments levels between 1409A and 1409B (Figure 3 and Table S1), except for lutein. The content of chlorophyll, carotenoid, anthocyanin and isoflavone in the etiolated leaves decreased by 57.0%, 58.9%, 64.6% and 61.3%, respectively, compared with those in the green plants. However, the etiolated leaf accumulated 13.3% higher the total content of flavone (Table S1). 

**Figure 4:**
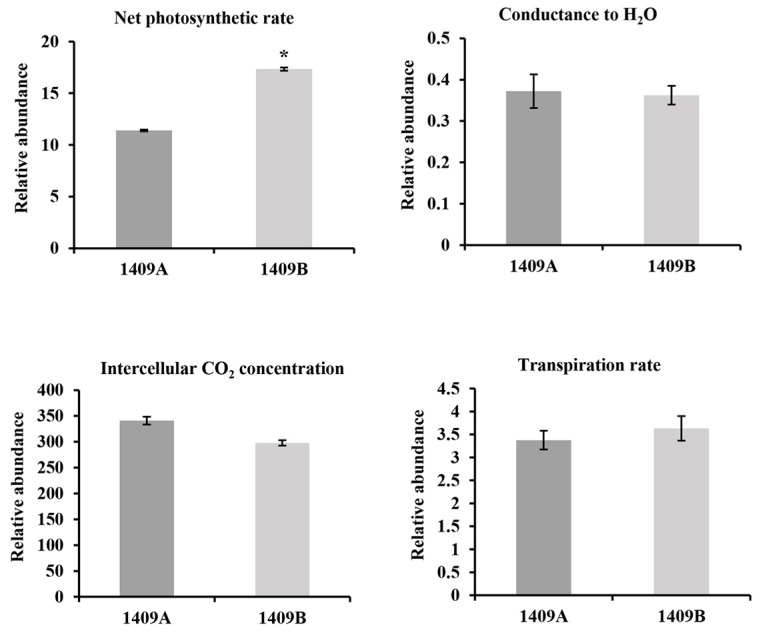
Analysis of the leaf net photosynthetic rate (Pn), conductance to H_2_O (Co.), intercellular CO_2_ concentration (Ci) and transpiration rate (Tr) in seedling leaves of 1409A and 1409B. Values are the means ± SE from three biological replicates (*n* = 3). Asterisks indicate the significant differences (* *p* < 0.01) between 1409A and 1409B as determined by Student’s *t* test.

We further analyzed the potential effect of photosynthesis on the etiolated leaves, and then measured the photosynthesis-related attributes in both lines of Chinese cabbage. The results indicated no differences in conductance to H_2_O (Co), intercellular CO_2_ concentration (Ci) and transpiration rate (Tr) between the etiolated and non-etiolated leaves. However, a remarkable difference in the net photosynthetic rate (Pn) was found: Pn in some of the etiolated leaves was decreased by 34.3% (Figure 4 and Table S2).

### 2.3. Transcriptome Assembly and Annotation

From the above mentioned physiological features and ultrastructure of chloroplasts, we assumed that the expression pattern of genes responsible for pigment biosynthesis and chloroplast development was changed in the 1409A line. To test our hypothesis and understand the molecular basis of etiolated leaves in the Chinese cabbage *hau* CMS line, we performed high-throughput sequencing using six leaf samples from the 4–6 true leaves stage. In total, 40.00 Gb of clean data were produced, with over 6.00 Gb clean data for each sample; more than 91.56% reads had a quality score of Q30 (sequencing error rate, 0.1%). All of the bases were distinguished, where the clean data GC content ranged from 47.74% to 48.62%. Each sample’s clean reads were aligned with a reference genome sequence, where the alignment efficiency was between 63.25% and 67.49% (Table S3). After directly comparing the gene expression levels in different samples, we found that both the sequencing quality and gene expression level were generally identical among these samples (Figure S1). These results indicate that the RNA-Seq data we obtained were of sufficiently high quality to warrant further analysis.

### 2.4. Identification of Differentially Expressed Genes (DEGs)

**Figure 5:**
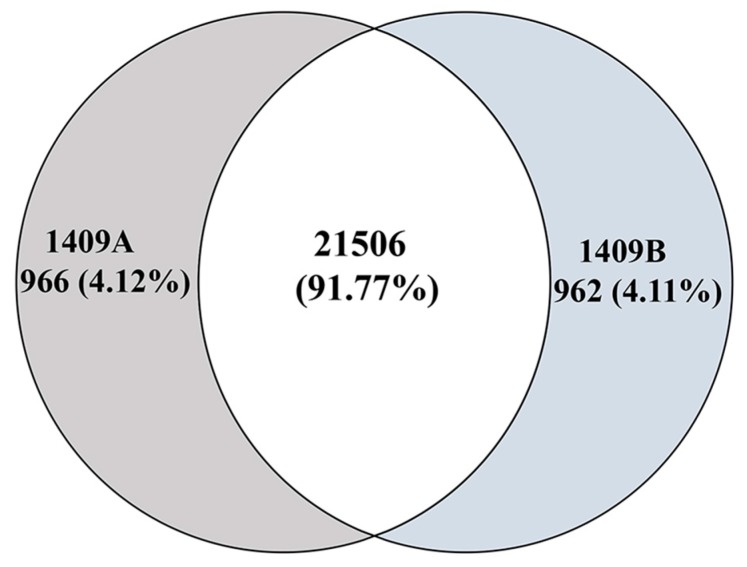
The number of specific genes and shared genes between 1409A and 1409B lines of Chinese cabbage.

**Figure 6:**
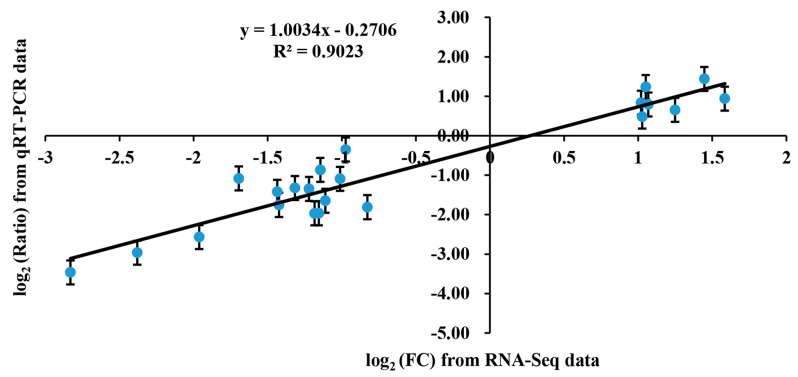
Coefficient analysis of fold-change data between qRT-PCR and RNA-Seq. Data indicating the relative transcript level as determined by qRT-PCR are the means of three replicates. Scatterplots are generated by log_2_ expression ratios from RNA-Seq (*x*-axis) and qRT-PCR (*y*-axis).

The expression level of the genes was calculated and normalized to FPKM (fragments per kilobase million). Consequently, 22,472 and 22,468 genes were identified, respectively, in the cDNA libraries from 1409A to 1409B leaves, and, of these identified genes, 966 and 962 genes were expressed specifically in the leaves of 1409A and 1409B, respectively (Figure 5). In this study, based on twofold changes and FDR (false discovery rate) <0.05 as the selection criteria, 485 DEGs were detected between 1409A and 1409B, including 189 up-regulated genes and 296 down-regulated genes. We used an MA (M, intensity ratio; A, average intensity) plot to compare the DEGs based on significant differences (Figure S2). To confirm the RNA-Seq data, we performed qRT-PCR assays using Chinese cabbage leaves at the same developmental stage as those used for DEG analysis (Table S4). As shown in Figure 6, the qRT-PCR data were consistent with the RNA-Seq data in terms of relative fold changes in the expression of 22 genes between 1409A and 1409B (Pearson correlation coefficient, 0.902). The qRT-PCR results basically verified the expression level of DEGs found in RNA-Seq and were consistent with the phenotypes of etiolated leaf in 1409A line. 

### 2.5. Functional Classification of DEGs

**Figure 7:**
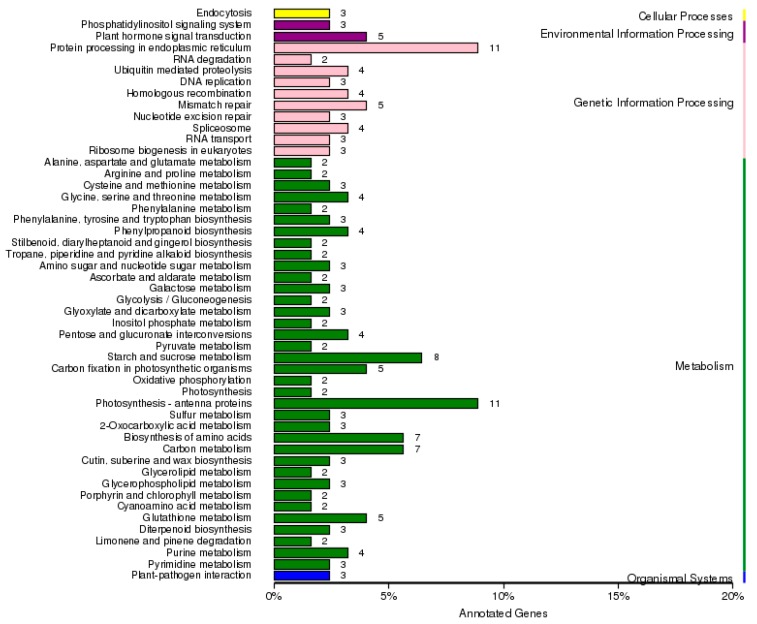
Classification statistics for differentially expressed genes in 1409A and 1409B according to KEGG pathway analysis. Cellular processes, environment information processing, genetic information processing, metabolism and organismal systems are expressed in yellow, purple, pink, dark green and blue, respectively.

In the present study, the predicted functions of 485 DEGs were obtained by GO (Gene Ontology) annotation, KEGG (Kyoto Encyclopedia of Genes and Genomes) pathway, and enrichment analyses. According to GO annotation, these DEGs were distributed into 40 functional terms as follows: 20 terms for biological process, 16 terms for cellular component and 17 terms for molecular function (Figure S3). The DEGs of topGO enrichment in the 10 biological process groups were mainly involved in organ morphogenesis (GO:0009887), regionalization (GO:0003002) and chlorophyll biosynthetic process (GO:0015995) (Figure S4a and Table S5). The DEGs of topGO enrichment in the 10 cellular component groups were mainly involved in intracellular part (GO:0044424), membrane-bounded organelle (GO:0043227) and chloroplast stromal thylakoid (GO:0009533) (Figure S4b and Table S5). The DEGs of topGO enrichment in the 10 molecular function groups were mainly involved in methyltransferase activity (GO:0008168), dipeptide transporter activity (GO:0042936) and tripeptide transporter activity (GO:0042937) (Figure S4c and Table S5). Pathway analysis based on KEGG revealed 82 metabolic pathways related to photosynthesis antenna proteins, protein processing in endoplasmic reticulum and starch and sucrose metabolism, all of which differed significantly between 1409A and 1409B leaves (Figure 7). 

Meanwhile, KEGG enrichment analysis showed that DEGs were significantly enriched in the pathways of diterpenoid biosynthesis, flavone and flavonol biosynthesis, and mismatch repair. Photosynthesis-related genes were specifically suppressed in the etiolated leaves, such as “photosynthesis antenna proteins”, “starch and sucrose metabolism”, “carbon metabolism”, and “carbon fixation in photosynthetic organisms” were mainly suppressed in the etiolated leaves (Table S6).

**Figure 8:**
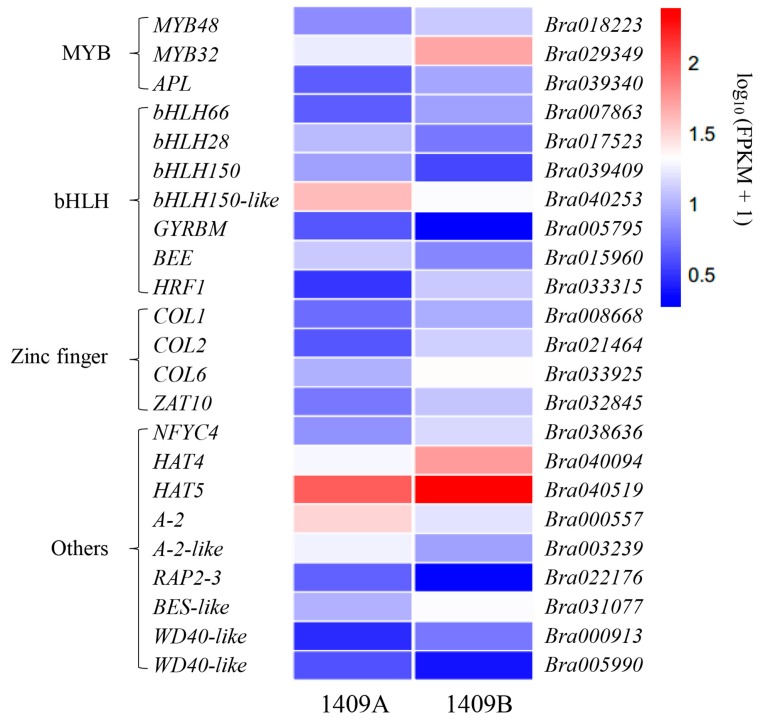
Expression model of MYB, bHLH and other TFs might involve in the regulation of anthocyanin and flavonoid metabolism. The value of log_10_ (FPKM + 1) is shown by a color gradient from low (blue) to high (red). Each column in the heat map represents 1409A or 1409B.

Moreover, 23 DEGs predicted to encode TFs were identified (Figure 8). These genes were divided into eight categories: MYB, bHLH, zinc finger, NF-YC (nuclear factor YC), HD-ZIP (homeodomain-leucine zipper), HSF (heat shock factors), WRKY (the WRKY domain with the conserved amino acid sequence WRKYGQK) factor, and ERF (ethylene response factor). Among these genes, 13 genes had significantly decreased transcript levels in 1409A leaves, while the expression of the remainder were significantly more expressed in 1409A. Genes annotated as *MYB48* (*Bra018223*), *MYB32* (*Bra029349*), *APL* (*Bra039340*), *bHLH66* (*Bra007863*) and *HFR1* (*Bra033315*) were down-regulated in 1409A, while the remaining genes predicted to encode MYB and bHLH were up-regulated (Table S7). Additionally, the expression of TFs annotated as zinc finger, NF-YC, HD-ZIP, HSF, WRKY, and ERF family members also changed significantly during seeding leaf development in 1409A (Table S7).

## 3. Discussion

The structure and quantity of chloroplasts in the etiolated leaves of 1409A were obviously distinct from those of 1409B. Moreover, the ultrastructure of chloroplasts from 1409A changed significantly (Figure 2), which showed a typical program of chloroplast–to-chromoplast transition as described by Vanegas-Espinoza et al. [40]. Correspondingly, leaf color results from the processes of pigment accumulation in the plastid, which includes chloroplast development and division, biosynthesis and transport of pigments, such as chlorophylls and carotenoids [16]. Carotenoids that accumulate in plastoglobules (PGs), which are thylakoid-associated lipid droplets, play an essential role in protection from excess light [41]. In addition to acting as photoprotective compounds, carotenoids also serve as precursors in the biosynthesis of several phytohormones [42]. In this study, the content of chlorophyll and carotenoid in the 1409A plants was significantly lower than that in the 1409B (Figure 3 and Table S1). The finding is consistent with the results of Chang et al. [43] regarding the alloplasmic lines of *B. rapa* with partly chlorotic leaves. Moreover, we found that *CYP707A4* (Figure 6 and Table S4), which is necessary for carotenoid biosynthesis [44], was down-regulated in 1409A. These results showed that the disturbance of chlorophyll biosynthesis or down-regulated expression of the *PORB* gene might be responsible for Pchlide homoeostasis and etiolated leaf [15].

Chlorophyll is essential for harvesting light energy during photosynthesis [45]. POR catalyzes the penultimate reaction of chlorophyll biosynthesis, i.e., the light-triggered reduction of protochlorophyllide to chlorophyllide [46]. *PORB* plays an important role in Pchlide homeostasis and greening of etiolated plants. Failure to express functional *PORB*, as demonstrated for the generated (Cys→Ala)-PORB proteins, led to photodynamic damage [15]. In the study, through RNA-Seq and biochemical analyses, we find that only the expression of *PORB* is down-regulated significantly in 1409A comparing to 1409B during chlorophyll synthesis. Therefore, we speculate that alteration in the expression of *PORB* may be responsible for the etiolated leaves.

Our experimental study demonstrated abnormally developed chloroplasts (Figure 2) and impaired photosynthesis (Figure 4 and Table S2) in the etiolated leaves, which were consistent with the results of KEGG classification of enriched pathways (Figure 7). Especially, feedback mechanisms are critical to the regulation of chloroplast development, and signals from functional plastids are required to maintain nuclear expression of genes encoding chloroplast proteins; for example, *Lhcb* was responsive to plastid signals from the mutant *GUN1* [47]. Our study found that 11 DEGs that control the expression of photosynthesis antenna proteins are down-regulated in the etiolated leaves (Table S6). In addition, the gene *rbcL*, encoding the large subunit of Rubisco, which is an enzyme involved in the first major step of carbon fixation, is down-regulated significantly in the etiolated leaves. This finding indicates that the pathways related to photosynthesis metabolism were mainly inhibited, which might be retrograde process controlled by organelle-to-nucleus signaling.

The cooperation of chloroplast genes and nuclear genes is necessary for normal development of chloroplasts of higher plants, which means that changes in the expression of either genome tend to affect the biogenesis of normal chloroplasts and that, consequently, the disruption in chlorophyll metabolism or chloroplast assembly can contribute to abnormal leaf color [16]. The *GLK* gene family, as previous studies have reported, acts as the crucial regulatory factor for chloroplast development in rice, *Arabidopsis thaliana* and tomato [22,23,48,49,50,51,52]. For rice, the partial expression of overlapping domains of *AtGLK1* and *AtGLK2* in insertion mutants resulted in single mutants that displayed normal leaf color, showing a degree of functional redundancy [52]. The analysis of the gun1-101 mutant showed that normal expression levels of *GLK1* mRNA failed to accumulate the GLK1 protein, which means that plastid signals directly regulate the accumulation of theprotein in a *GUN1*-independent manner [48]. Mutant and transgene analyses in tomato demonstrated that a latitudinal gradient of *GLK2* expression influences the typical uneven coloration of green and ripe wild-type fruit [22,23]. In line with these research findings, the 1409B plants developed mature chloroplasts compared to the 1409A plants, which did not have distinctly complete thylakoid structures (Figure 2). Meanwhile, the expression levels of *GLK1* and *GLK2* in the leaves of 1409B plants were higher than those in the 1409A. Hence, our research has further proven that the expression level of the *GLK* gene is closely related to chloroplast development in various plant species.

Structural plant genes are usually regulated by TFs, but we found few studies identified TFs regulating chlorophyll and carotenoid biosynthesis and degradation. Those studies were mostly performed in model plants [53,54]. However, no TFs regulating chlorophylls and carotenoids were screened in our study. A growing body of evidence suggests that anthocyanin and flavonoid biosynthesis in plants is controlled by a transcription complex, the MYB-bHLH-WD40 (MBW) complex, composed of the R2R3-MYB, bHLH and WD40 proteins [28,55,56]. Moreover, the content of anthocyanin, flavone and isoflavone significantly differed between 1409A and 1409B in this study (Figure 3 and Table S1).

In plants, anthocyanins and flavonoids are the main pigments and play an important role in many biological processes. For example, anthocyanins serve as photoprotective light screens in vegetative tissues [57], flavonoids are available for UV-scavenging, fertility and disease resistance [58]. The transcript abundances of *MYB114* and *MYB10* were correlated, and the co-transformation of these two genes into tobacco and strawberry led to enhanced anthocyanin biosynthesis [59]. Previously, genes such as *MYB12* were shown to play a positive role in the regulation of flavonoid biosynthesis in kale (*Brassica oleracea* var. *acephala*) [60]. In our study, we found the *MYB32*, *MYB48* and *ALP* showed a significant decrease in the expression and that *MYB305* had an up-regulated profile (Figure 8). Thus, these genes may play an important role in seedling etiolated leaves in *B. rapa*.

The bHLH proteins function as anthocyanin biosynthesis regulators and have been reported in model plants and vegetable species, including *Arabidopsis* [61], tobacco [62,63], red cabbage [64], tomato [65], and Chinese cabbage [66]. These proteins usually serve as co-factors interacting with R2R3-MYBs to induce anthocyanin biosynthesis. In this study, five bHLHs had much lower expression levels in etiolated leaves, and the other bHLHs had higher expression levels (Table S7). This result suggests that the co-expression of bHLH and MYB may induce abnormal anthocyanin biosynthesis in etiolated leaves of Chinese cabbage. In this article, through RNA-Seq analyses, we found that up-regulated expression *TT7* (Figure 6 and Table S4), which is thought to be imperative for flavonoid biosynthesis [67], tends to lead to increased flavone accumulation and reduced isoflavone content (Figure 3 and Table S1). The role of *TT7* gene in etiolated leaf need to be further verified.

## 4. Materials and Methods

### 4.1. Plant Materials

The *hau* CMS was originally found as a spontaneous male sterile mutant in *B. juncea* in the experimental field of Huazhong Agricultural University. The *B. juncea* CMS line (*hau* CMS) was crossed with the Chinese cabbage (*B. rapa*) followed by multiple backcrosses using Chinese cabbage as the recurrent parent. Finally, the eighth generations (BC_8_) line were selected to establish the Chinese cabbage *hau* CMS system. The plants for backcrossing were selected based on two major characteristics: phenotypic similarity to the recurrent parent Chinese cabbage with leaf etiolation and male sterility. The Chinese cabbage *hau* CMS line 1409A and its corresponding maintainer line 1409B, were seeded in 50-hole aperture disks with normal nursery substrate in a greenhouse facility of the National Center of Vegetable Improvement, Huazhong Agricultural University, with a natural photoperiod and 22/18 °C day/night temperature during the 2015–2016 cropping season. When the plants grew to 3–5 true leaves at the seedling stage, which showed obvious etiolation of the leaves, newly emerged leaves were collected from 1409A to 1409B plants. Immediately after harvest, samples were frozen in liquid nitrogen and stored at −80 °C until analysis.

### 4.2. Assays of the Content of Main Pigment

Approximately 1 g fresh leaves from 1409A to 1409B plants was cut into pieces and then submerged in 20 mL extraction buffer (acetone:ethanol = 8:1, *v*/*v*) at room temperature in the dark for 24 h. Then, the contents of chlorophyll and carotenoid in the leaf extract were measured by specific light absorption at 470, 645 and 663 nm using a TU-1810D UV-Visible spectrophotometer (Beijing Purkinje General Instrument Co., Ltd., Beijing, China) [68]. Anthocyanin was extracted with 0.1 mol/L acidic alcohol for 30 min in a 60 °C water bath, and then 5 mL of acidic alcohol was added at room temperature to extract for 15 min twice. The samples were analyzed by specific light absorption at 665, 649, and 470 nm using the improved procedure described previously with some modifications [69,70]. Following the procedure of Hentschel et al. [71] with some modifications, anhydrous ethanol was used to extract lutein in an ultrasound bath, and then, the lutein pigments in the leaf extract were determined at 446 nm. The isoflavone extraction was performed on the basis of the method of Glencross et al. [72] with some modifications. Approximately 0.5 g of leaves were submerged in 30 mL of 70% alcohol for 2 h in a 60 °C water bath. The mixture was centrifuged at 5000 rpm for 15 min, the supernatant was collected, and the absorbance was measured at 243, 263 and 283 nm. The flavone content was analyzed using the ethanol method as described by Kosalec et al. [73] with a slight alteration. Approximately 0.3 g of leaves from 1409A to 1409B plants were ground into powder, followed by reflux extraction twice with 80% alcohol; then, the filter was evaporated to dryness and dissolved in 10 mL of 30% alcohol. The absorbance of the mixture was determined at 520 nm, and the concentration of flavone was obtained in the standard working curve with rutin as the standard sample. All measurements were performed with three biological replicates. Data were analyzed using Microsoft Excel 2013 (Microsoft Corporation, Redmond, WA, USA), and statistical tests were analyzed by Student’s *t* test using the SPSS 19.0 program (SPSS Inc., Chicago, IL, USA).

### 4.3. Determinations of Photosynthetic Rate

Photosynthesis measurement systems can be used to measure the photosynthetic rate. These systems measure the photosynthetic rate using an infrared gas analyzer to assess the input of CO_2_ and output of H_2_O. In this study, a portable photosynthesis machine LI-6400XT (LI-COR Inc., Lincoln, NE, USA) was used for determining the photosynthetic rate in Chinese cabbage on sunny, windless days from 9:00 to 11:00 a.m. following the procedure described previously [74]. Leaf temperature was controlled at 25 °C, and photon flux density was maintained at 500 µmol m^−2^ s^−1^. The net photosynthetic rate (Pn), conductance to H_2_O (Co), intercellular CO_2_ concentration (Ci) and transpiration rate (Tr) were recorded on fully outstretched leaves of the second youngest nodes. Young leaves were selected randomly with three biological replicates.

### 4.4. Transmission Electron Microscopy

Leaf discs 1.0 × 2.0 mm in size were prepared from 3–5 true leaves stage leaves dissected from 1409A to 1409B plants. After a prefixation procedure in 4% glutaraldehyde for 24 h at 4 °C followed by 1% OsO_4_ for 2 h, tissues were dehydrated through an acetone series. The samples were embedded in Epon 812 and sectioned into 50–80-nm-thick slices using a Leica UC6 ultramicrotome (Leica Microsystems, Ltd., Wetzlar, Germany). After the samples were stained with 2% (*w*/*v*) uranyl acetate and 2.6% (*w*/*v*) lead citrate, the ultrastructure of the leaf cells was examined under a Hitachi H-7650 transmission electron microscope (Hitachi Science Systems, Ltd., Tokyo, Japan) following the method of Cao et al. [75].

### 4.5. RNA Extraction, cDNA Library Construction, and Sequencing

Six samples from three biological replicates of 1409A and 1409B plant leaves exhibiting a significantly etiolated and green phenotype at the seedling stage were applied for transcriptomic analysis. Total RNA from each sample was extracted according to the instruction manual of TRIzol reagent (Life Technologies Corporation, Carlsbad, CA, USA). RNA integrity and concentration were checked using an Agilent 2100 Bioanalyzer (Agilent Technologies, Inc., Santa Clara, CA, USA). A total amount of 1 μg RNA per sample was used as input material for the RNA sample preparations. 

Sequencing libraries were generated using a NEBNext Ultra^TM^ RNA Library Prep Kit for Illumina (New England Biolabs, Ipswich, MA, USA) following the manufacturer’s recommendations, and index codes were added to attribute sequences to each sample. In brief, the enriched mRNA was fragmented into approximately 200 nt RNA inserts, which were used to synthesize the first-strand cDNA and second-strand cDNA. The double-stranded cDNA then underwent end-repair/dA-tail and adaptor ligation. The suitable fragments were isolated by Agencourt AMPure XP beads (Beckman Coulter, Inc., Brea, CA, USA) and enriched by PCR amplification. Finally, the constructed cDNA libraries were sequenced on a flow cell using an Illumina HiSeq™ 2500 sequencing platform; sequencing was performed by Beijing Biomarker Biotechnology Co., Ltd. (Beijing, China). The data of RNA-seq were deposited in the NCBI’s Sequence Read Archive and the accession number is SRP127386.

### 4.6. RNA-Sequencing Data Analysis

Raw reads were filtered to obtain high-quality reads by removing low-quality reads containing adaptors, unknown nucleotides > 5%, or Q20 < 20% (percentage of sequences with sequencing error rates < 1%) by a Perl script. The clean reads that were filtered from the raw reads were mapped onto the *B. rapa* genome using TopHat 2.1.1 (Daehwan Kim and Steven Salzberg at Johns Hopkins University, and Cole Trapnell at the University of Washington) [76]. The reference genome can be download at the website: http://brassicadb.org/brad/datasets/pub/Genomes/Brassica_rapa/V1.0/V1.5/. Gene expression levels were estimated using FPKM values by the Cufflinks software (Cole Trapnell’s lab at the University of Washington) [77].+ 

Differential expression was estimated and tested with the software package DEseq (R version: 3.1.1) (Simon Anders, EMBL Heidelberg, Germany) [78]. We calculated gene abundance differences between those samples based on the fold change (FC) of the FPKM values. FDR control method was used to identify the threshold of the *P*-value in multiple tests to compute the significance of the differences. The genes with a FDR significance score <0.05 and an absolute value of log_2_ (FC) ≥ 1 were determined to be significantly differentially expressed (DEGs).

The gene sequences were further aligned to the Clusters of Orthologous Group (COG) database to predict and classify functions [79]. Gene Ontology (GO) annotations and Kyoto Encyclopedia of Genes and Genomes (KEGG) pathway analyses were conducted using Blast2GO (v2.5) software (BioBam, Valencia, Spain) [80]. GO enrichment analysis of the DEGs was implemented by the GOseq R packages based on the Wallenius non-central hyper-geometric distribution [81]. Additionally, The KOBAS (v2.0) (Center for Bioinformatics, Peking University) software was utilized to test the statistical enrichment of the DEGs in KEGG pathways [82]. 

### 4.7. Quantitative Real-Time PCR Analysis 

The total RNA of 1409A and 1409B samples used for the RNA-Seq analysis were also used for qRT-PCR validation. The single-stranded cDNAs used for qRT-PCR were synthesized from 5 µg total RNA with a PrimeScript RT-PCR Kit (TaKaRa, Dalian, China). The qRT-PCR was performed using a SYBR Premix Ex Taq II Kit (TaKaRa, Dalian, China) on a LightCycler^®^ 96 Real-Time PCR System (Roche, Basel, Switzerland). A three-step program was used, with an initial hot start at 95 °C for 30 s, followed by 40 cycles of 95 °C for 5 s and 58 °C for 34 s. Melting curves were generated using the following program: 95 °C for 15 s, 60 °C for 1 min, and 95 °C for 15 s. A list of sequence-specific primers used for qRT-PCR including the *Actin* gene and the 22 selected genes is displayed in Table S1. Three technical replicates were carried out for each sample, and the relative expression levels were normalized to the expression of the *Actin* gene and calculated using the 2^−ΔΔCt^ method [83].

## 5. Conclusions

**Figure 9:**
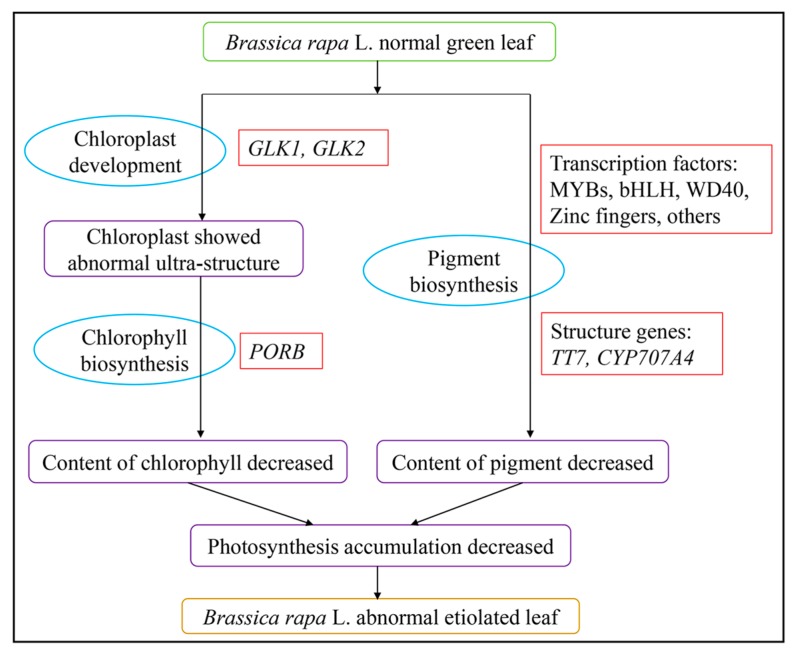
Possible formation pathway of etiolated leaves of the *Brassica rapa* L. line 1409A.

The analysis of the ultrastructure of chloroplasts and of physiological characteristics indicated that obvious differences between chloroplasts from 1409A to 1409B existed. RNA-Seq analysis verified DEGs involved in chloroplast development and division, chlorophyll biosynthesis and pigment biosynthesis. The DEGs between 1409A and 1409B were identified by qRT-PCR. In view of the above-mentioned results, we conclude that leaf etiolation in the 1409A line was caused by the following mechanism: abnormal chloroplast development directly or indirectly influenced chlorophyll and pigment biosynthesis, leading to distinct contents of chlorophyll and pigment and, finally, giving rise to leaf etiolation (Figure 9).

## Supplementary Materials

Supplementary Materials can be found at http://www.mdpi.com/1422-0067/19/4/922/s1.
