# Supplementary material for: Transcriptome Analysis Reveals Candidate Genes Associated with Leaf Etiolation of a Cytoplasmic Male Sterility Line in Chinese Cabbage (Brassica Rapa L. ssp. Pekinensis)
Source: Int J Mol Sci. 2018 Mar 21;19(4):922. doi: 10.3390/ijms19040922 (PMC5979472; doi:10.3390/ijms19040922)
Supplement: Supplementary file 1 [file ijms-19-00922-s001.pdf]

Supplementary Tables

Table S1. Content of main pigments in leaves of 1409A and 1409B at the seedling stage.

Pigments	1409A	1409B
Chlorophyll a + b (mg·g−1 FW)	0.882 ± 0.022	2.050 ± 0.077*
Carotenoid (mg·g−1 FW)	0.166 ± 0.006	0.374 ± 0.144*
Anthocyanin (μg/g)	1.290 ± 0.051	3.648 ± 0.087*
Flavone (mg/g)	2.215 ± 0.009*	1.955 ± 0.005
Isoflavone (μg/g)	43.836 ± 0.315	113.408 ± 2.084*
Lutein (%)	0.011 ± 0	0.012 ± 0

* Significant 1% probability according to the Student's *t* test.

Table S2. Net photosynthetic rate (Pn), conductance to H2O (Co), intercellular CO2 concentration (Ci) and transpiration rate (Tr) in leaves of 1409A and 1409B at the seedling stage.

Photosynthesis	1409A	1409B
Net photosynthetic rate (Pn) (μmol CO_2 m^{-2} s^{-1})	11.396 ± 0.079	17.340 ± 0.153*
Conductance to H_2O (Co) (mol H_2O m^{-2} s^{-1})	0.372 ± 0.041	0.363 ± 0.023
Intercellular CO_2 concentration (Ci) (μmol CO_2 mol^{-1})	341.124 ± 7.452	297.680 ± 5.216
Transpiration rate (Tr) (mmol H_2O m^{-2} s^{-1})	3.376 ± 0.205	3.634 ± 0.267

* Significant 1% probability according to the Student's *t* test.

Table S3. Summary of the transcriptome assembly. 1409A, T04-T06; 1409B, T01-T03.

BMK-ID	Total Reads	Mapped Reads	Uniq Mapped Reads	Multiple Map Reads	Reads Map to '+'	Reads Map to '-'
T01	47,873,118	32,308,246 (67.49%)	31,400,578 (65.59%)	907,668 (1.90%)	15,923,108 (33.26%)	15,864,961 (33.14%)
T02	44,330,020	29,035,221 (65.50%)	27,460,616 (61.95%)	1,574,605 (3.55%)	13,978,237 (31.53%)	13,934,086 (31.43%)
T03	40,481,396	25,603,159 (63.25%)	24,581,695 (60.72%)	1,021,464 (2.52%)	12,482,074 (30.83%)	12,432,111 (30.71%)
T04	43,590,384	28,566,675 (65.53%)	27,768,772 (63.70%)	797,903 (1.83%)	14,084,798 (32.31%)	14,031,152 (32.19%)
T05	46,664,810	30,510,420 (65.38%)	29,698,325 (63.64%)	812,095 (1.74%)	15,052,234 (32.26%)	15,000,412 (32.15%)
T06	47,218,238	30,899,128 (65.44%)	30,036,585 (63.61%)	862,543 (1.83%)	15,229,945 (32.25%)	15,167,959 (32.12%)

Table S4. Sequence-specific primers used for qRT-PCR

Gene	Annotation	Primer-F (5'-3')	Primer-R (5'-3')
	Actin	GGAGCTGAGAGATTCCGTTG	GAACCACCACTGAGGACGAT
Bra019049	PORB1	AAAGCGTCTCATTATCGTCG	CTGTTTAAGCCGTTCAATCC
Bra033574	ROC1	CGGGAAGGATCGTGATGGAG	GTGGAGGGGCTTTCCCTTAC
Bra040519	ATHB-1	CCAGCAGCAGCTTCATCC	GGTGCGTCGTCTTCGTCTA
Bra022407	CYP707A4	AACCATCCGTAAACTCATTCC	TCTCCCTGTAAGAACCCTCC
Bra030303	FBA1	GCATTGACAGGACATACGAGG	TCAGCACCAGGAGTAACCAT
Bra000708	LHCB5	GGCGTCTATTGGTGTTTCG	TGGCAGGAGCTGGCTTT
Bra029349	MYB32	CCGGGATCTACTAACCGGGA	CACCGTTTTGAGCAGTGTGG
Bra033315	HFR1	AGAGCAAGAAGTGATAAAGAACG	CATCAGGGACAGCCAACG
Bra033925	COL6	GGAAAGACGGCGAGGGC	GGCAAGTGGGTTGGCTGA
Bra004491	CYP709B2	TCTACTGGCAAGGAACAGAGC	TGACGAACCCAATCAATACC
Bra015784	AMY2	TTGGCTTCGTTGGTTGC	ACGGTTTCGCTGCTCCT
Bra012371	GGT1	TATCCACTCTACTCAGCCACCA	CCATTGCCCTTACTGATATTCC
Bra014635	BGL2	ATAACCTACCAAAGCCACCG	GGGAACGTCGAGGATGAAC
Bra036015	GLK2	TGATGAACAAGAAATGGAGGGA	GTTTTCCTCTACGCACCATTTT
Bra031129	GKL1	CACATCATACGGTGTACAAACC	CGTCAATACATCTCCTATGGCT
Bra009312	TT7	GCCCGCACTTGATTGTTT	CTTCATCGCCTCGTGTTCT
Bra040253	bHLH150	CTCGGCGAAGCACAACG	CGAACCGGCTCACCAATAT
Bra000557	ATHSFA2	CATAGATGATGAGGCGAGTGA	CAACGGTGAGGCTACCAAA
Bra007237	At5g15810	GCTACACGAGGACAGGAACTAT	CAGGGACATCCCAACAGAC
Bra007327	PCMP-H81	GGAAACGGGCAAGACGC	TCCTTCATCCACCATCCCT
Bra031484	At1g60770	CATTGTATGGACGGCTTGG	TGAACAGTGTCTCCGCACC
Bra029677	HSP83A	ATGGGTATTGCTCTTTCCG	ATCCACTTCGACTGGCTCA

Table S5. The top 10 most represented GO terms of DEGs in three groups

GO.ID	Term annotation	All genes	DE genes	Expected	KS
**Biological Process**					
GO:0048041	focal adhesion assembly	21	0	0.27	7.00E-06
GO:0042891	antibiotic transport	16	0	0.21	0.00018
GO:0016068	type I hypersensitivity	12	0	0.16	0.00025
GO:0009855	determination of bilateral symmetry	602	5	7.82	0.00037
GO:0003002	regionalization	1896	22	24.63	0.00046
GO:0009887	organ morphogenesis	2779	39	36.11	0.00066
GO:0042138	meiotic DNA double-strand break formation	223	1	2.9	0.00074
GO:0042939	tripeptide transport	81	5	1.05	0.00099
GO:0016925	protein sumoylation	79	1	1.03	0.00115
GO:0015995	chlorophyll biosynthetic process	499	9	6.48	0.00135
**Molecular Function**					
GO:0090353	polygalacturonase inhibitor activity	14	0	0.19	0.00031
GO:0000175	3'-5'-exoribonuclease activity	34	1	0.45	0.00055
GO:0008705	methionine synthase activity	12	1	0.16	0.00067
GO:0080039	xyloglucan endotransglucosylase activity	31	1	0.41	0.00129
GO:0070401	NADP+ binding	13	0	0.17	0.00151
GO:0008168	methyltransferase activity	642	16	8.53	0.00155
GO:0033946	xyloglucan-specific endo-beta-1,4-glucanase activity	34	1	0.45	0.00167
GO:0004675	transmembrane receptor protein serine	97	2	1.29	0.00173
GO:0047807	cytokinin 7-beta-glucosyltransferase activity	18	0	0.24	0.00175
GO:0080062	cytokinin 9-beta-glucosyltransferase activity	18	0	0.24	0.00175
**Cellular Component**					
GO:0005634	nucleus	15872	171	196.38	0.0032
GO:0031463	Cul3-RING ubiquitin ligase complex	38	1	0.47	0.0048
GO:0009921	auxin efflux carrier complex	11	1	0.14	0.0049
GO:0044424	intracellular part	30942	382	382.83	0.0051
GO:0005815	microtubule organizing center	22	1	0.27	0.0071
GO:0009506	plasmodesma	4587	77	56.75	0.0087
GO:0005775	vacuolar lumen	31	1	0.38	0.0089
GO:0005694	chromosome	471	13	5.83	0.0091
GO:0043227	membrane-bounded organelle	28634	354	354.28	0.0104
GO:0009533	chloroplast stromal thylakoid	40	2	0.49	0.0115

Table S6. KEGG pathway enrichment of DEGs between 1409A and 1409B

ko_id	*Kegg_pathway	Unigene number	Gene number	P-value
ko00196	Photosynthesis - antenna proteins	11	34	1.87E-12
ko00904	Diterpenoid biosynthesis	3	37	0.020
ko04141	Protein processing in endoplasmic reticulum	11	359	0.0252
ko00944	Flavone and flavonol biosynthesis	1	2	0.0312
ko03430	Mismatch repair	5	116	0.0356
ko00073	Cutin, suberine and wax biosynthesis	3	52	0.0479

* Kegg_pathway with the threshold of P-value < 0.05 was listed.

Table S7. MYB, bHLH and other transcription factors regulate the synthesis of anthocyanin.

Term	Gene	Annotated function	FPKM	
			1409A	1409B
MYB	Bra018223	MYB48	11.942	6.203
	Bra029349	MYB32	49.877	17.402
	Bra039340	APL	7.997	3.713
bHLH	LOC103943771	bHLH66	7.705	3.523
	LOC103945359	bHLH28	4.837	9.995
	LOC103950478	bHLH150	2.821	7.759
	LOC103957537	bHLH150-like	19.886	40.122
	LOC103958164	GYRBM	0.888	3.510
	LOC103938518	BEE	5.794	11.595
	LOC103941022	HRF1	12.094	2.340
Zinc Finger	LOC103946790	COL1	8.794	4.353
	LOC103966388	COL2	12.570	3.470
	LOC103931693	COL6	20.857	9.103
	LOC103941900	ZAT10	11.207	4.884
Others	LOC103944179	NFYC4	13.632	6.626
	LOC103950248	HAT4	54.219	18.688
	LOC103952761	HAT5	239.053	97.485
	LOC103952763	A-2	15.026	30.673
	LOC103953943	A-2-like	7.786	18.003
	LOC103958416	RAP2-3	1.066	3.955
	LOC103967196	BES-like	19.565	8.932
	LOC103933485	WD40-like	4.950	2.047
	LOC103937070	WD40-like	1.437	3.132

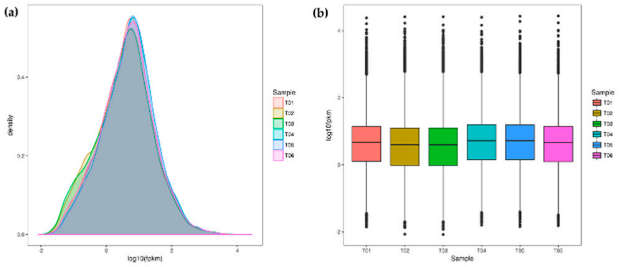

Figure S1. The FPKM density and boxplot FPKM distribution of each sample. **(a)** The different color curves denote different samples, the horizontal axis indicates corresponding sample log10(FPKM), and the vertical axis indicates corresponding probability density; **(b)** The horizontal axis represents different samples, the vertical axis represents the sample log10(FPKM). 1409A, T04-T06; 1409B, T01-T03.

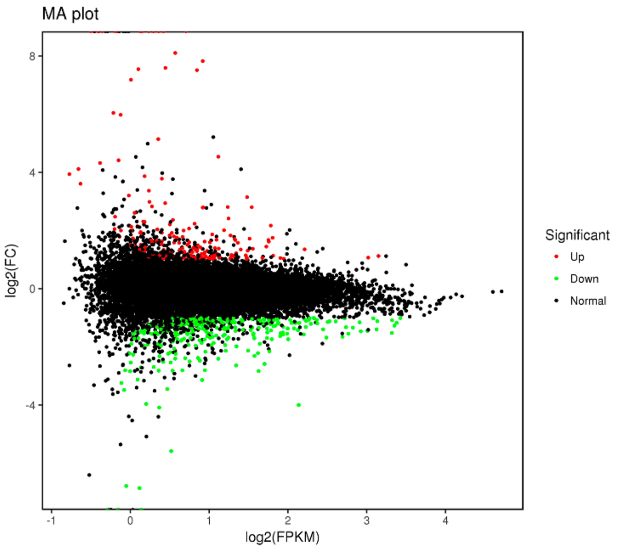

Figure S2. The gene expression of the two samples and the overall distribution of the fold changes in an MA plot. Each point in the MA map represents a single gene. The x-axis is log2 (FPKM), that is the value of the mean of expression in two samples, the y-axis is log2(FC), that is the number of differences in gene expression between two samples, which is used to measure the differences of the expression amount. The green dots, the red dots and the black dots represent down-regulated differentially expressed genes and up-regulated differentially expressed genes and non-differentially expressed genes, respectively.

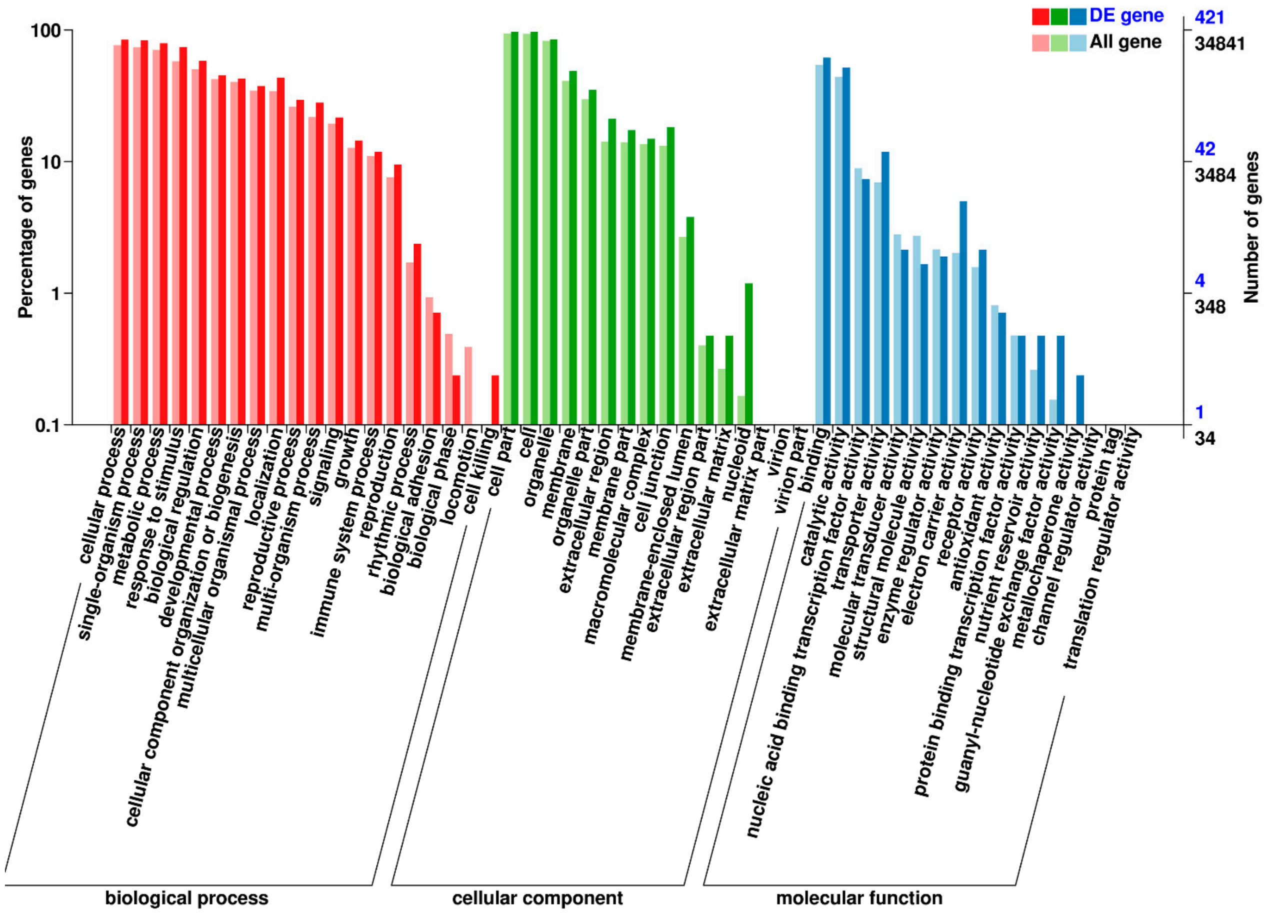

Figure S3. Classification statistics for DEGs in 1409A relative to 1409B according to the GO annotations. The x-axis is the GO classification, and the y-axis is the percentage of the number of genes, the right is the number of genes. The GO classification was represented using different colors, with red representing biological process, green representing cellular component and blue representing molecular function.

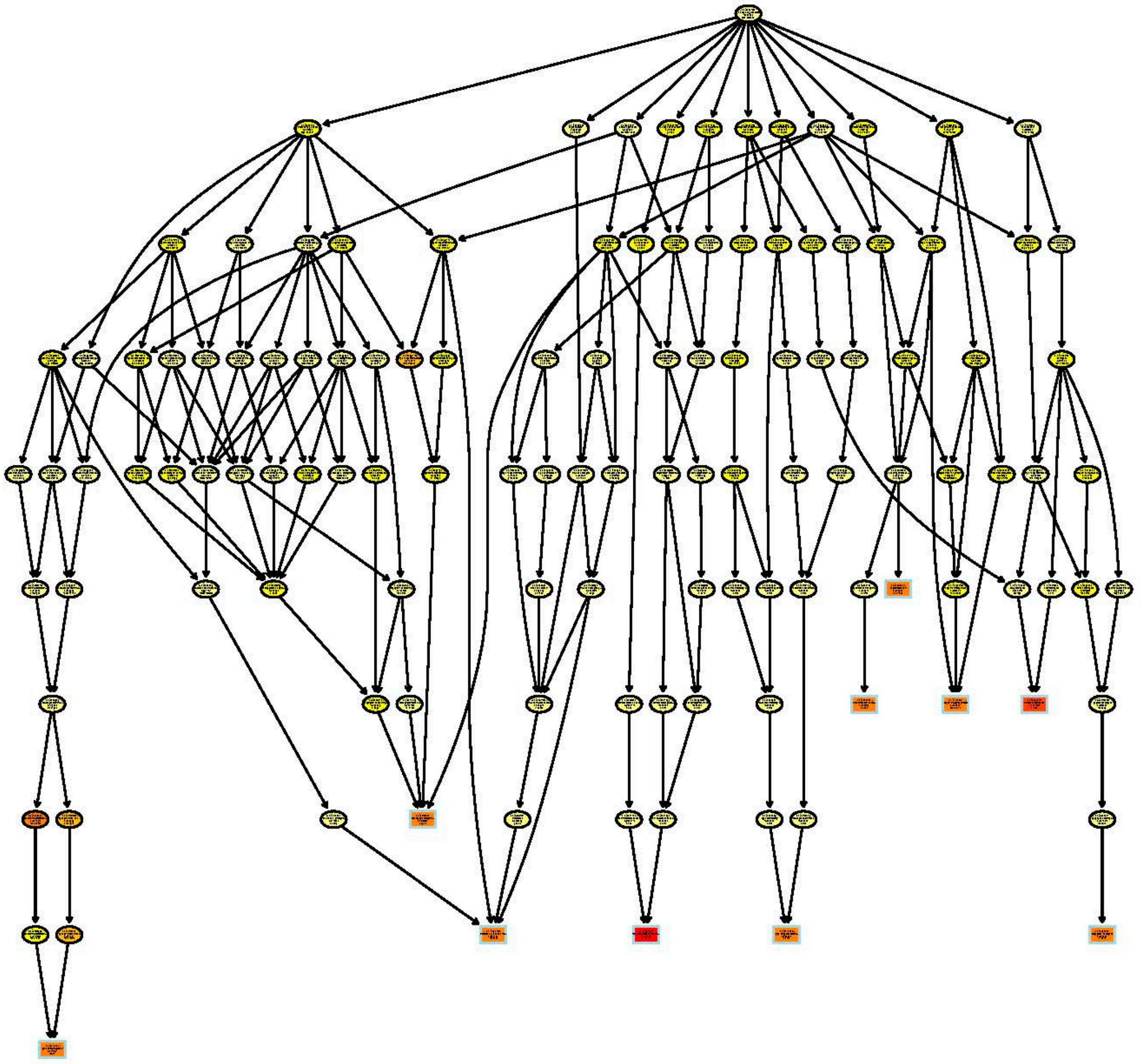

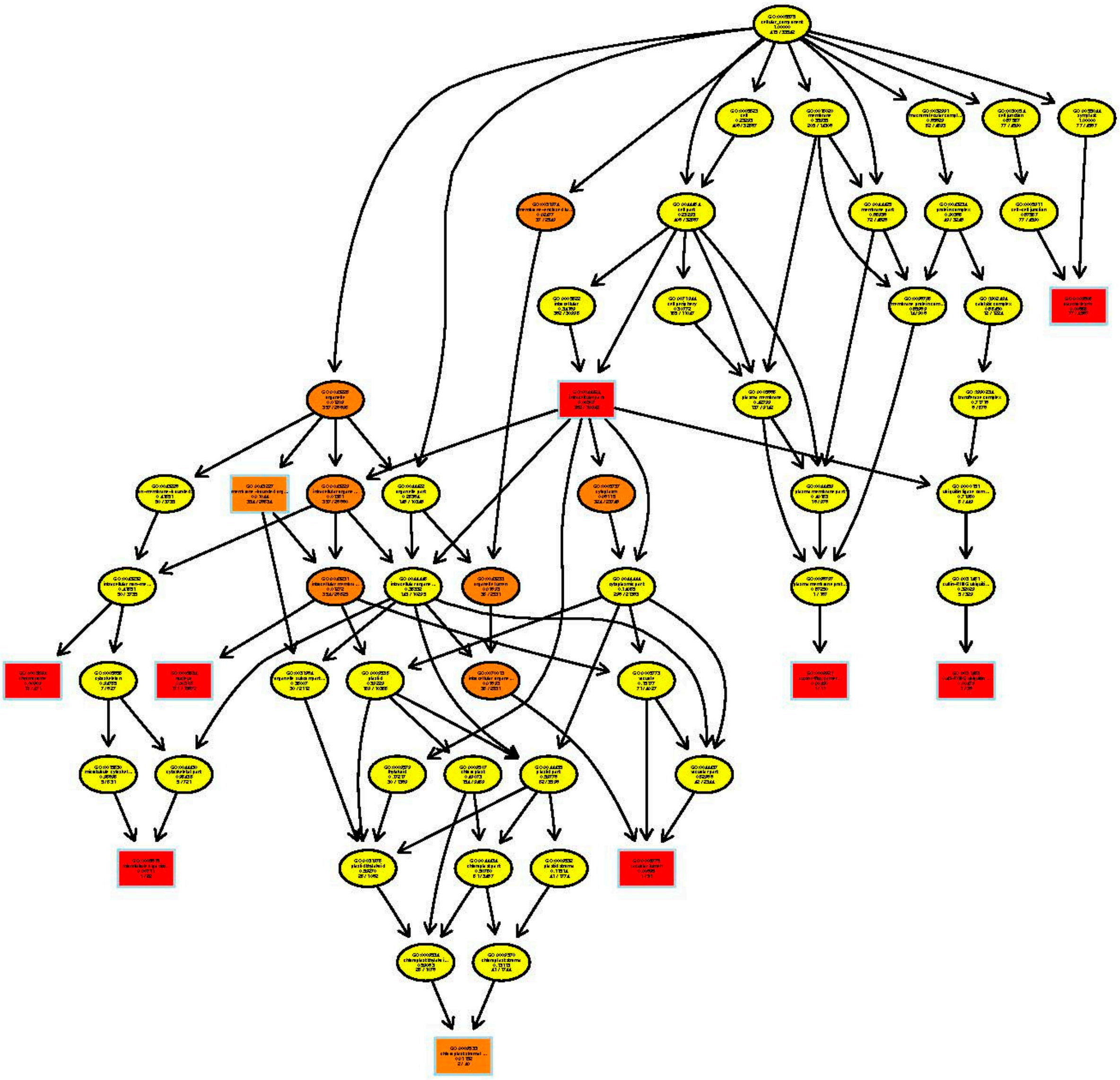

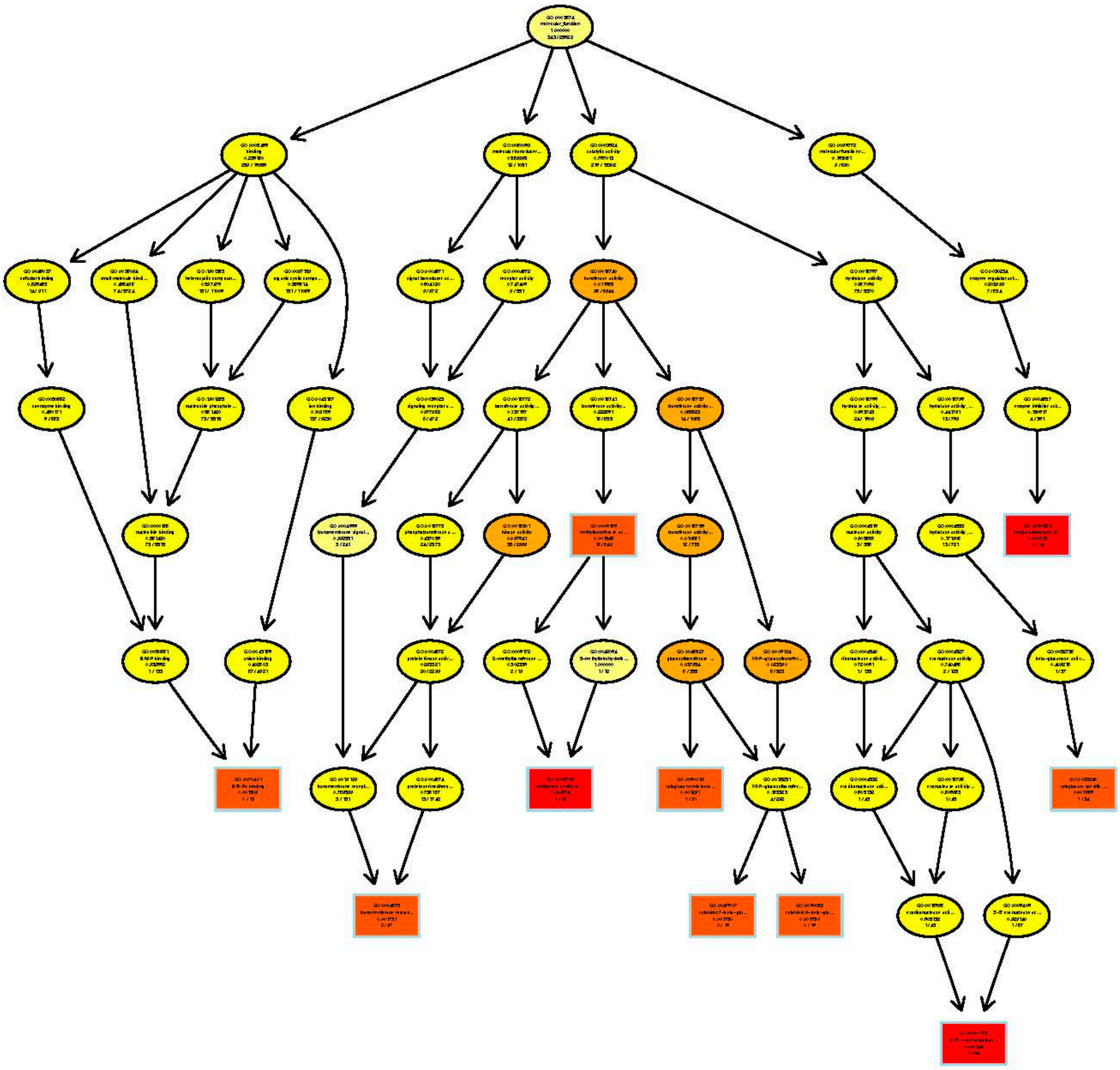

Figure S4. The DEGs of topGO enrichment in three GO categories. **(a)** The DEGs of topGO enrichment in the 10 biological process groups; **(b)** The DEGs of topGO enrichment in the 10 cellular component groups; **(c)** The DEGs of topGO enrichment in the 10 molecular function groups. The content description and enrichment significance of this GO node are given in each box (or ellipse). Different colors represent different enrichment significance, the deeper the color, the higher the significance.
